# An integrated baseline assessment of reef-associated sharks around Saba (Dutch Caribbean), combining three methods: stereo-BRUVs, telemetry and citizen science

**DOI:** 10.1098/rsos.241754

**Published:** 2025-07-09

**Authors:** Guido Leurs, Martin de Graaf, Dahlia Hassell-Knijff, Larissa Ayumi Kuramae Izioka, Wouter van Looijengoed, Melanie P. Meijer zu Schlochtern, Tadzio Bervoets, Leopold A. J. Nagelkerke, Hendriks V. Winter

**Affiliations:** ^1^ Marine Animal Ecology Group, Wageningen University and Research, Wageningen, Gelderland, The Netherlands; ^2^ Wageningen Marine Research, IJmuiden, Noord-Holland, The Netherlands; ^3^ Saba Conservation Foundation, The Bottom Saba, Bonaire, Sint Eustatius and Saba; ^4^ Dutch Caribbean Nature Alliance, Bonaire, Bonaire, Sint Eustatius and Saba; ^5^ Caribbean Shark Coalition, Kralendijk, Bonaire, Sint Eustatius and Saba; ^6^ Aquaculture and Fisheries Group, Wageningen University and Research, Wageningen, Gelderland, The Netherlands

**Keywords:** site fidelity, residency, acoustic telemetry, Caribbean reef shark, Atlantic nurse shark, marine protected area

## Abstract

The Exclusive Economic Zone of Saba (Dutch Windward Islands) has been designated Yarari Marine Mammal and Shark Sanctuary. However, to effectively conserve sharks, a baseline on their diversity and spatiotemporal distribution is required. We used three methods: telemetry, stereo-baited remote underwater video systems (stereo-BRUVs) and a citizen science program to study reef-associated sharks, mainly Caribbean reef sharks (*Carcharhinus perezi*) and nurse sharks (*Ginglymostoma cirratum*) around Saba during 2012–2020. Based on the stereo-BRUVs, we determined that the presence of Caribbean reef sharks (0.25 ± 0.48 maxN h^−1^; mean ± s.d.) and nurse sharks (0.20 ± 0.45 maxN h^−1^) was relatively high compared to other Caribbean coral reef systems. With telemetry, we showed that the residence and fidelity of Caribbean reef sharks were high, especially for immature individuals. In addition, we showed a higher presence of Caribbean reef sharks during December–May and a lower overall presence of adult males. The citizen science program showed an increase in observations of both species between 2012 and 2020, possibly indicating an increase in abundance or a change in shark behaviour related to the culling of exotic lionfish. We demonstrate that these three methods can be used complementary to provide a baseline of the diversity and spatiotemporal presence of reef-associated sharks.

## 1. Introduction

Over 30% of all shark species are currently threatened with extinction [1]. Shark populations worldwide experience declines in abundance and diversity caused by the combined effects of overfishing [1,2], habitat degradation or destruction [1,3,4] and the vulnerability of these species [5]. Due to the many shark species having K-selected life-history characteristics (e.g. slow growth, late maturity and low productivity) their populations experience low intrinsic growth, which makes this species group more susceptible to exploitation and slower to recover compared to most teleost fishes [6–8].

Declines of predatory fishes such as sharks can negatively impact ecosystems, as these species fulfill various roles within the marine food web as both meso- and top-predators, translocate nutrients between multiple ecosystems and sustain ecosystem services [9–12]. The removal of predatory fishes like reef sharks from coral reef ecosystems may affect coral cover and the overall health of coral reef ecosystems through cascading effects [13–15].

Within the Greater Caribbean region, reef-associated shark populations and coral cover declined over the past decades due to increased fishing pressure and habitat degradation [3,16,17]. Within the Western Atlantic region, shark populations have declined by 76–89% [2,18], and hard coral cover within the Greater Caribbean has decreased by 80% in just over two decades (1980–2003) [19,20]. These declines and understudied connections between the status of predatory fishes and coral cover highlight the importance of improved ecosystem-based management strategies within the Wider Caribbean.

Since September 2015, the Exclusive Economic Zones (EEZ) of Saba and Bonaire (Dutch Caribbean) have been designated as the Yarari Marine Mammal and Shark Sanctuary [21]. In 2018, this sanctuary was extended to include the EEZ of Sint Eustatius. However, for the effective conservation of marine species, a baseline survey assessing the status, residency and site fidelity of reef-associated sharks is essential to evaluate the success of management actions in protected areas [22–25]. Moreover, the potential cross-boundary movements of reef-associated sharks might also call for implementing a network of interconnected marine protected areas (MPAs) [26]. Information on the spatiotemporal habitat use and site fidelity of reef-associated sharks can increase the effectiveness of MPAs and no-take zones to conserve vulnerable shark species [24,27]. In addition, data on temporal diel activity, seasonal habitat use and ontogenetic habitat shifts [28] can further improve these management efforts by preventing interference with human activities [29]. Baseline data on the diversity, relative abundance and spatiotemporal distribution of reef-associated sharks like the Caribbean reef shark (*Carcharhinus perezi*) and the nurse shark (*Ginglymostoma cirratum*) is currently limited to Dutch Caribbean waters. This study aims to provide an integrative approach to establish baseline data for these species in the coastal waters of Saba, Dutch Caribbean. In addition, this study aims to describe how three commonly used field methods to study sharks (i.e. baited remote underwater video, acoustic telemetry and visual census) can be deployed effectively in a complementary manner for tracking vital indicators on shark population status and to assess management effectiveness in the future.

## 2. Material and methods

### 2.1. Study area

**Figure 1.**
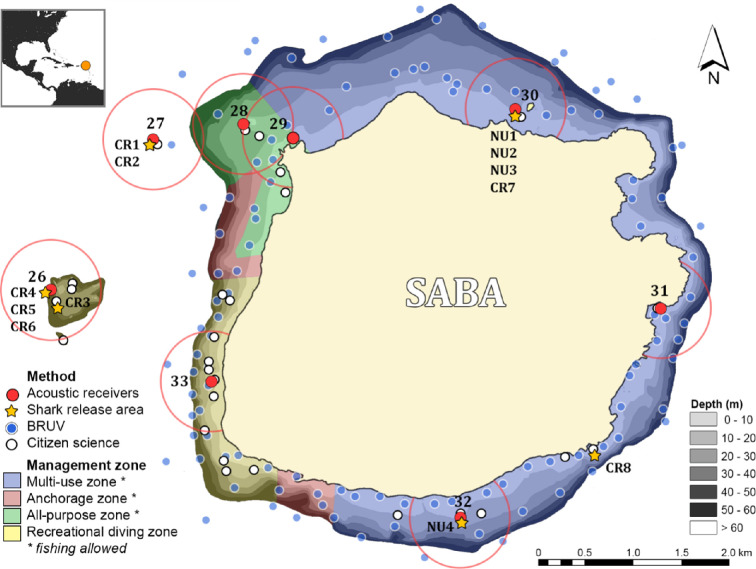
Overview of the study area and sample locations for each method. Locations of acoustic receivers are indicated in red with associated detection ranges (± 500 m) and identification numbers (26–33). Capture and release locations are indicated for all sharks included in the acoustic telemetry study (CR1−9 for Caribbean reef sharks and NU1−4 for nurse sharks). Citizen science dive sites are indicated in white, and BRUV-sample sites are indicated in blue. Depth contours (0–60 m) are indicated in greyscale, and the different management zones are indicated in blue (multi-use zone), red (anchorage zone), green (all-purpose zone) and yellow (recreational diving).

The island of Saba (17°37′54.3″ N, 63°14′15.8″ W) is located in the Northeastern Caribbean Sea (figure 1). Saba is a small (13 km^2^) volcanic island with high terrestrial elevation. Underwater, the bathymetry consists of steep sea walls, which causes the sea bottom to drop to depths larger than 60 m. Off the island’s West coast, several pinnacles (i.e. seamounts) rise to a depth of ±30 m under the surface. The Saba National Marine Park (13 km^2^) extends from the high tide level to a depth of 60 m all around the island and includes a marine reserve (4.29 km^2^) consisting of four different zones: the multi-use zone (i.e. accommodates boating and diving, but less emphasis on dive tourism), anchorage zone (i.e. area specific for anchoring of vessels), all-purpose zone (i.e. emphasis of recreation by means of boating, swimming, diving and fishing) and recreational diving zone (i.e. emphasis on diving, only surface trolling fisheries allowed) (figure 1).

### 2.2. Data collection

#### 2.2.1. Stereo-BRUV

Three stereo-baited remote underwater video (s-BRUV) systems designed as described by Langlois *et al*. [30] were deployed from July to December 2012. Each system consisted of an aluminium frame with two cameras (Canon Legria HFG10) in PVC housings mounted in a horizontal plane relative to the sea bottom (70 cm apart, converged 8° inward). A synchronizing diode in the focal area of both cameras enabled the cameras to be synchronized before data analysis. Each stereo-BRUV set-up was calibrated with SeaGIS CAL v.2.01 (www.seagis.com.au). Each set-up was baited with approximately 800 g of pilchards (*Sardinops* sp.) and was deployed in shallow (±15 m) and mesophotic (±50 and ±100 m) habitats. The distance between BRUV consecutive deployments was a minimum of 500 m to reduce the probability of sharks moving between sampling sites [31]. Each BRUV was deployed for a minimum of 60 min.

#### 2.2.2. Citizen science

In 2012, a citizen science project was initiated in cooperation with the dive guides of the Sea Saba dive school. During each scuba dive, an experienced diver recorded the species and number of individuals (including zero observations), the time of day, the dive location and the maximum depth attained during the dive. Dives occurred on 29 different dive sites distributed around the entire island and generally lasted between 40 and 50 min. Observations from 2012 to January 2021 were included in this study. Due to the change in ownership of the dive school, no data were available from 2021 to 2023. We used observations from 2024 for comparison of shark observation rates between dives with and without culling of invasive lionfish (*Pterois volitans*) as this was the only complete year of data collection on lionfish culling (data collection started mid-2023). Lionfish culling was not conducted on tourist dives prior to 2021 due to local regulations.

No shark provisioning is done by dive operators in the waters around Saba.

#### 2.2.3. Acoustic telemetry

In October 2014, eight InnovaSea VR2W acoustic receivers were installed around the island, covering a large area of its coastal waters and offshore pinnacles (figure 1). Acoustic receivers were placed in open, sandy locations using two concrete blocks (approx. 15 kg) as the substrate anchor and a subsurface buoy to ensure the receiver remained in a vertical position. In October 2014, 12 sharks—eight Caribbean reef sharks and four nurse sharks—were captured with rod and reel, longlines or as bycatch in lobster traps. Sharks were caught using circle hooks. Upon capture, sharks were inverted to induce a trance-like state called ‘tonic-immobility’ to reduce stress levels during the procedure [32]. InnovaSea v.16 transmitters (69 kHz, approx. 1713 day battery life) were surgically implanted in the abdominal cavity through a 2 cm incision on the ventral side along the midline of the body. The incision was closed with two or three stitches using dissolvable braided nylon sutures (Surgilon). Transmitters were programmed to emit a unique acoustic signal every 80 s, with random intervals of 50–110 s, to prevent collision of multiple acoustic signals. Depending on environmental conditions, the detection range of these transmitters by a receiver can be up to 600 m in tropical conditions [33]. Prior to release, total length (TL, to the nearest cm) and sex were recorded. Throughout the tagging procedure, fresh seawater was poured over the sharks to oxygenate the gills. The sharks were released within 15 min.

### 2.3. Data analysis

#### 2.3.1. Stereo-BRUV

To limit recounting of the same individual during a deployment, shark densities were measured as the maximum number of individuals of a species in one frame per deployment of 60 min (MaxN) [34]. The fork length of each shark was measured to the nearest centimetre using the SeaGIS EventMeasure software package (www.seagis.com.au). Only sharks approaching within 8 m of the stereo-BRUV were measured to ensure accurate length estimates (e.g. [35]). In addition, the influence of habitat complexity, depth and management zones were determined and tested for significance using a chi-square test. Habitat complexity was scored using the six-point scale of Polunin & Roberts [36], ranging from 0 (no vertical relief) to 5 (exceptionally complex due to coral cover). In total, 110 stereo-BRUV deployments were conducted at depths of approximately 15 m (*n* = 56), approximately 50 m (*n* = 31) and approximately 100 m (*n* = 23). For analysis, these deployments were grouped in shallow (approx. 15 m) and deep (approx. 50 and approx. 100 m pooled) to achieve similar sample sizes. The four different management zones of the Saba National Marine Park were also included in the analysis (figure 1).

#### 2.3.2. Citizen science (scuba diving)

The proportion of dives with at least one shark present was calculated for the Caribbean reef and nurse shark separately. In addition, the sightings-per-unit-effort (SPUE) was calculated for each dive site by dividing the mean number of sharks observed by the number of dives. Differences in SPUE were tested for significance using a Mann–Whitney *U*-test.

The presence of both species was analysed using a logistic generalized linear mixed model with a logit-link function. Fixed terms for the initial model included habitat depth in metres (i.e. measured as the maximum attained depth during a dive), season (dry season: December–April; wet season: May–November), year, light conditions (day: 06.00–17.59; night: 18.00–05.59), and management zone (i.e. fishing permitted or zones where fishing is not allowed; figure 1). An interaction term between depth and management zone was also included, and the dive site was included as a random effect. Model selection was conducted based on the Akaike information criterion (AIC) and the Bayesian information criterion (BIC).

We used the 2024 citizen science data for a comparison of shark observations between dives with and without lionfish culling. To do this we tested if the number of individual sharks differed between across dives with and without culling using a Mann–Whitney *U-*test due to the non-normal distribution of shark count data. We also tested whether the proportion of dives with the presence of sharks was higher during culling compared to dives without culling. For this, we used a binomial generalized linear model with shark presence during a dive as a response and lionfish culling as a dependent variable.

#### 2.3.3. Acoustic telemetry

The site fidelity of each individual was determined by calculating the detection indices (i.e. the number of days detected at a site divided by the days at liberty, the period from capture to the end of the study period). Residency was determined and defined as the longest period of consecutive days that an animal was present within the study area.

Seasonal and diurnal patterns of presence were determined only for Caribbean reef sharks, due to the availability of data. For this, only sharks that were detected for more than 50 days were included, resulting in a sample size of six individuals. The presence/absence of this species was analysed using a generalized additive mixed model with a binomial distribution. Predictor variables included the hour of the day and month. To account for the cyclical nature of these variables, a cyclical smooth was applied to these variables. The individual shark and year were included as random effects. The best-fit model was selected based on AIC and BIC values.

## 3. Results

### 3.1. Stereo-BRUV

**Figure 2.**
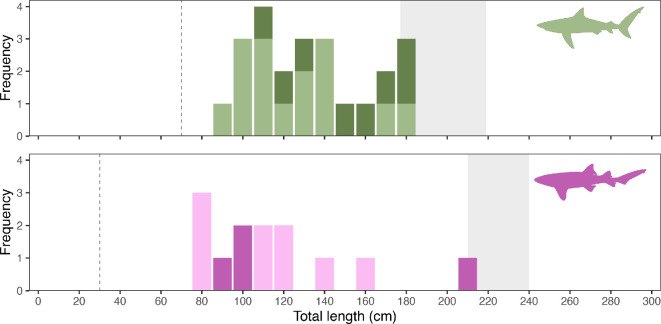
Observed size frequency distribution for Caribbean reef sharks (top, green) and nurse sharks (bottom, purple). Frequencies are indicated for sharks observed on the baited remote underwater video systems (light green/light purple) and sharks captured during the acoustic telemetry study (dark green/dark purple). Dashed lines indicate size at birth, and grey areas indicate mixed-sex size-at-maturity range.

A total of 28 sharks of five different species were recorded, with the Caribbean reef shark and nurse shark accounting for the highest number of observations (table 1). The other three species were the great hammerhead shark (*Sphyrna mokarran*, *n* = 1), blacktip shark (*Carcharhinus limbatus*, *n* = 1) and silky shark (*Carcharhinus falciformis*, *n* = 1). The length of the Caribbean reef sharks ranged from 79 to 259 cm, with a mean size of 116 ± 44 cm (figure 2). The size of nurse sharks ranged from 65 to 132 cm, with a mean size of 93 ± 21 cm (median: 96 cm).

**Table 1.** Effort for each different field method. dep.: deployments; obs.: observations; div.: dives; det.: detections.

method	Caribbean reef shark	nurse shark	total	sampling days
	(*Carcharhinus perezi*)	(*Ginglymostoma cirratum*)		
stereo-BRUV			110 (dep.)	19
observed (*n*)	17	9	26 (obs.)	
citizen science			10 728 (div.)	2624
observed (*n*)	6581	3057	9638 (obs.)	
acoustic telemetry				1733
tagged (*n*)	8	4	12 (sharks)	
detections (*n*)	660 784	29 907	690 691 (det.)	

**Figure 3.**
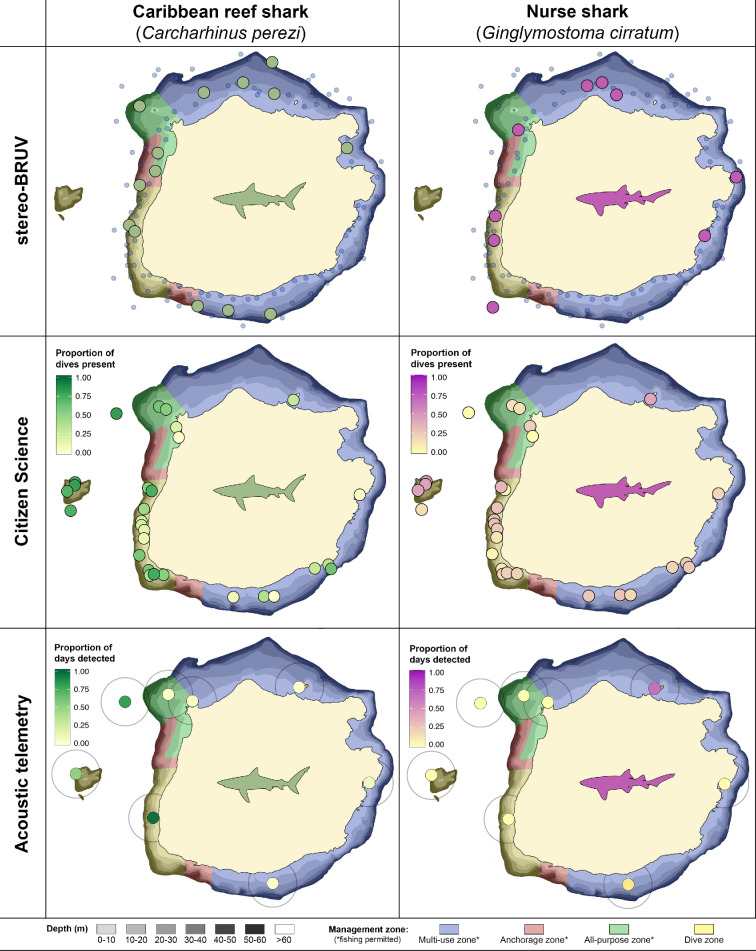
Spatial distribution of the Caribbean reef shark (*Carcharhinus perezi*) and nurse shark (*Ginglymostoma cirratum*) around Saba based on the three methods. The presence of both species based on stereo-BRUV (top) is indicated in green (Caribbean reef shark) and purple (nurse shark), blue circles indicate deployments with no observations of these species. The proportion of dives observed during the citizen science in green-gradient for Caribbean reef sharks and purple-gradient for nurse sharks (middle). The proportion of days detected within the acoustic telemetry array during the study period for both species (bottom). Depth contours are indicated in greyscale, and management zones are in blue (multi-use zone), red (anchorage zone), green (all-purpose zone) and yellow (recreational dive zone).

Most Caribbean reef sharks were observed in shallow (approx. 15 m) habitats (0.25 ± 0.48 MaxN h^−1^; mean ± s.d.) and at 50 m depth (0.24 ± 0.50 MaxN h^−1^) (figure 3). Nurse sharks were mainly observed in shallow habitats (0.20 ± 0.45 MaxN h^−1^). Only one individual was observed in deep habitats (approx. 100 m; figure 3). The number of Caribbean reef sharks was dependent on habitat complexity (χ^2^ = 10.4, d.f. = 4, *p* = 0.03), with most reef sharks occurring in habitats of moderate complexity (two on the scale of Polunin & Roberts [36]). Nurse sharks occurred in all habitats with a complexity >0, but the abundance of this species was not dependent on habitat complexity (χ^2^ = 3.8, d.f. = 4, *p* = 0.44). The abundance of Caribbean reef sharks was significantly higher in the management zone where fishing is not permitted (χ^2^ = 4.8, d.f. = 1, *p* = 0.03) compared to the three management zones where fishing is allowed. There were no significant differences in nurse shark abundance between the different management zones (χ^2^ = 2.8, d.f. = 1, *p* = 0.10). Deployments of stereo-BRUV systems were restricted to the habitats surrounding the island, resulting in a lack of data for the pinnacles located west of Saba (figure 3).

### 3.2. Citizen science (scuba diving)

**Figure 4.**
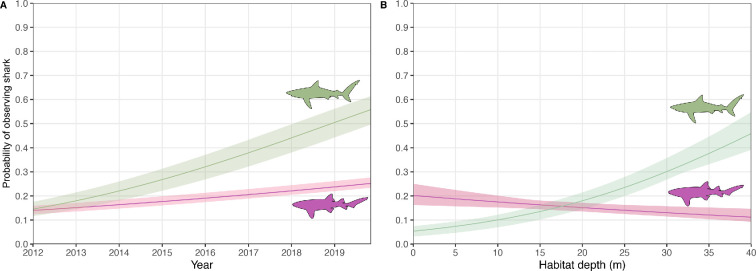
Predicted probability of observing Caribbean reef sharks (green) and nurse sharks (purple) for each study year (A) and with increasing habitat depth (B) as derived from the citizen science data (scuba diving). 95% confidence intervals are indicated for both species. Note that both trends reflect shark observations that can have different underlying factors (e.g. increased shark abundance or lionfish culling; see table 2).

**Figure 5.**
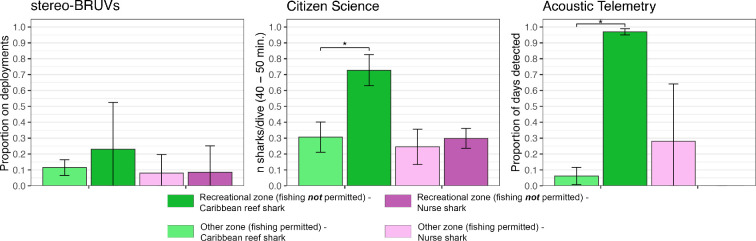
Relative presence of sharks using the three methods. Left: stereo-BRUV results are given as the number of sharks observed per hour for shallow and deep deployments combined. Centre: sightings per unit effort for citizen science data are given as the number of sharks per hour. Right: the proportion of days that at least one individual of a species was detected in the acoustic array. Results for Caribbean reef sharks are shown in green, and results for nurse sharks are shown in purple. Results for the recreational zone are indicated in dark green/purple, and results for other management zones are in light green/purple. Error bars show two times the standard error, and asterisks indicate significant differences.

During the 9 year study period, 10 728 dives were logged during 2624 days at sea (table 1). Depth ranged from 1 to 38.7 m, and dives were distributed across all months and 9 years of the study. Dives occurred in zones where fishing is permitted (*n* = 2565) and where fishing is not allowed (*n* = 8163). During 5619 dives, at least one reef-associated shark was observed, with the number of sharks per dive ranging from 0 to 9 individuals. In total, 9686 shark observations were recorded, with 68.0% of these observations being Caribbean reef sharks and 31.5% of the observations being nurse sharks. Other shark species (0.5%) that were observed were: blacktip sharks (*n* = 40) hammerhead sharks (*Sphyrna* spp.*, n* = 5), tiger sharks (*Galeocerdo cuvier*, *n* = 1) and silky sharks (*n* = 2). The highest presence of Caribbean reef sharks was measured on the dive sites around the pinnacles (observed during 61–73% of dives), followed by the other sites on the western side of Saba (figure 3). Around these pinnacles, the presence of nurse sharks was also highest (up to 43% of dives), together with a dive site on the north coast of Saba (41% of dives). The presence of Caribbean reef sharks on dive sites was best explained by year, season, habitat depth, light regime and management zone (electronic supplementary material, tables S1, S2). For nurse sharks, the same variables significantly explained their presence, except for the management zone (electronic supplementary material, tables S1, S2). The presence of both species during dives around Saba increased significantly over the past years (figure 4A). The observation probability of Caribbean reef sharks increased from 0.15 in 2012 to 0.56 in 2020 (β = 1.8, *z* = 25.1, *p* < 0.01), and of nurse sharks from 0.14 to 0.25 from 2012 to 2020 (β = 1.2, *z* = 8.6, *p* < 0.01). The probability of seeing Caribbean reef sharks increased significantly with habitat depth (β = 0.98, *z* = −1.99, *p* < 0.01), whereas the probability of seeing nurse sharks significantly decreased with depth (β = 1.1, *z* = 8.4, *p* = 0.046) (figure 4B). More Caribbean reef sharks per dive were observed on sites within the zone where fishing is prohibited (W = 41, *p* = 0.01), whereas there was no significant difference in observations of nurse sharks between the different management zones (figure 5).

The observation rate of both shark species was lower in 2024 compared to predicted observation rates. Caribbean reef sharks were observed on 43.0% of all dives and nurse sharks on 47.1% (table 2). The number of Caribbean reef sharks observed during lionfish culling dives (mean: 0.89 sharks per dive) was significantly higher compared to dives with no culling (0.66 sharks per dive; W = 55351, *p* = 0.03). Although the presence of this species was higher during dives with lionfish culling (mean = 50% of dives) compared to non-culling dives (42% of dives), this difference was not significant (*z*-value = 1.89, *p* = 0.06). For nurse sharks both the presence and the number of sharks observed did not differ significantly (table 2).

**Table 2.** Influence of invasive lionfish culling on diver shark observations during the citizen science program in 2024. Results are given for the number of sharks observed ('count') and the proportion of dives that sharks were present ('presence'). The total dives column indicates the number of dives during which no lionfish culling took place (*n* = 953), lionfish culling was conducted (*n* = 130) and the total number of logged dives in 2024 (*n* = 1083). The overall mean count and presence of the two shark species are also provided with a 95% confidence interval.

species	metric	lionfish cull	total dives (*n*)	mean (95% CI)	*p*‐value
Caribbean reef shark	count	no	953	0.66 (0.59–0.72)	
		yes	130	0.89 (0.68–1.09)	0.03
	presence	no	953	0.42 (0.39–0.45)	
		yes	130	0.50 (0.42–0.60)	0.06
		overall	1083	0.43 (0.39–0.51)	
nurse shark	count	no	953	0.74 (0.68–0.80)	
		yes	130	0.58 (0.45–0.71)	0.20
	presence	no	953	0.47 (0.44–0.51)	
		yes	130	0.45 (0.36–0.53)	0.55
		overall	1083	0.47 (0.43–0.51)	

### 3.3. Acoustic telemetry

**Figure 6.**
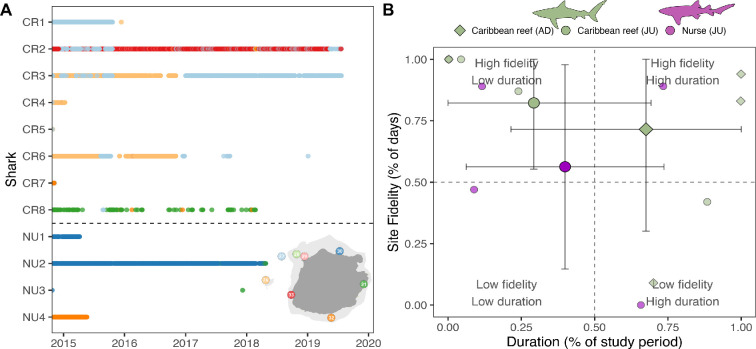
Detection patterns (A) and duration/site fidelity correlation (B) for the individual Caribbean reef sharks and nurse sharks based on acoustic telemetry. (A) Colours and numbers on the *y*-axis correspond with a specific location around the island. (B) The correlation between duration (i.e. the time between first and last detection expressed as a proportion of total study duration) and site fidelity (i.e. proportion of days that an individual was detected) is given for juvenile (circles) and adult (diamonds) Caribbean reef sharks (green) and juvenile nurse sharks (purple circles). The mean and standard error are shown for each group.

**Figure 7.**
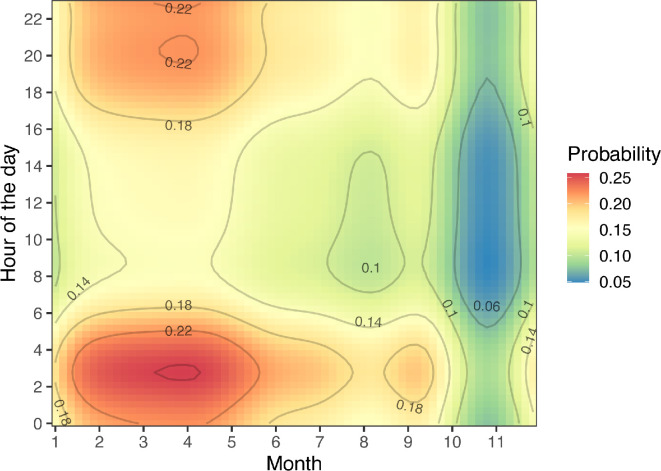
Predicted presence of Caribbean reef sharks around the island of Saba based on acoustic telemetry. Colours indicate the predicted probability (blue = low, red = high) for each combination of the month (*x*-axis) and hour of the day (*y*-axis). For this analysis, only sharks with more than 50 detections were used (*n* = 6 sharks).

The eight Caribbean reef sharks included in this study ranged from 115 to 184 cm TL (150.3 ± 27.8 cm; mean ± standard deviation) and included four males and four females (figure 2; table 3). The period of detection (i.e. ‘duration’, from first to last detection) of this species ranged from 4 to 1732 days (838.8 ± 789.8 days), with two individuals being detected within the last month of the study (table 3). Proportionally, Caribbean reef sharks were detected during more days within the recreational zone where fishing is not allowed (figure 5). Residency ranged from 2 to 317 days (136.5 ± 136.2 days), with the highest residency occurring around the pinnacles (figure 6). Adult Caribbean reef sharks were detected at more locations (4.5 ± 1.7 locations) compared to juveniles (2.5 ± 0.6 locations) (table 3). Individuals tagged in the vicinity of the pinnacles (locations 26 and 27 in figure 6A) showed high site fidelity (detection index (DI) > 0.75) to these sites (table 3). Intraspecific variation in site fidelity and presence was high and led to non-significant differences between life stages (figure 6B), but generally, site fidelity was higher for juvenile Caribbean reef sharks, whereas adult sharks had a higher presence in the waters of Saba (figure 6B). Caribbean reef shark presence showed a diurnal and seasonal pattern (electronic supplementary material, tables S3, S4), with generally a higher presence during night-time hours (17.00 to 06.00), but also a seasonally higher presence from January to June with the predicted detection probability in these months to be double the probabilities for other months (figure 7).

**Table 3.** Overview of the Caribbean reef sharks (*n* = 8) and nurse sharks (*n* = 4) tagged with acoustic transmitters. TL: total length; life stage: juvenile (JU) or adult (AD); loc.: location of tagging; duration: the period from first to last detection; Residency: the longest period of consecutive days detected within the receiver array; *n* rec.: number of receivers by which an individual was detected; site fidelity: detection indices (DI) within the total study area (DI_tot_), at the primary location (DI_1_) and secondary location (DI_2_).

	shark	sex	TL (cm)	life stage	loc.	detections (*n*)	duration (days)	residency (days)	*n* rec.	DI_tot_.	DI_1_	DI_2_
Caribbean reef	CR1	M	150	JU	27	1 44 287	416	317	2	0.87	0.87	<0.01
	CR2	F	184	AD	27	1 05 098	1732	269	7	0.94	0.92	0.19
	CR3	F	180	AD	26	3 37 467	1730	269	4	0.83	0.50	0.34
	CR4	F	131	JU	26	42 165	77	39	3	1.00	1.00	0.04
	CR5	M	115	JU	26	51	4	2	2	1.00	0.81	0.27
	CR6	F	125	JU	26	31 111	1532	179	3	0.42	0.38	0.04
	CR7	M	178	AD	30	24	4	2	4	1.00	0.71	0.71
	CR8	M	163	AD	32	581	1215	15	3	0.09	0.09	<0.01
nurse	NU1	F	94	JU	30	1133	154	7	1	0.47	0.47	
	NU2	F	105	JU	30	25 465	1273	97	3	0.89	0.89	<0.01
	NU3	F	210	JU	30	4	1140	1	3	0.00	<0.01	<0.01
	NU4	M	104	JU	32	3305	201	27	1	0.89	0.88	

The length of the four tagged nurse sharks ranged from 94 to 210 cm TL (128.3 ± 54.7 cm) and were all classified as three juvenile females and one juvenile male (figure 2; table 3). The period that nurse sharks were detected ranged from 154 to 1273 days (692.0 ± 596.9 days), with none of the individuals detected during the last month of the study (figure 6A). Nurse shark residency ranged from 1 to 97 days (33.0 ± 44.1 days). Site fidelity of these individuals was highest on the northern side of the island (figure 6A) but was lower compared to Caribbean reef sharks (figure 6B).

## 4. Discussion

We combined three different monitoring methods to determine the spatiotemporal distribution and assess the status of reef-associated sharks, which were mainly Caribbean reef sharks and nurse sharks. All three methods combined yielded a complementary and consistent picture of the distribution and presence of these shark species. We show that these species are common in the waters around Saba and that the observations of these species have increased throughout the past decade. Although some individuals may show high fidelity to specific sites (e.g. the pinnacles) or have a high presence in Saba’s territorial waters, intraspecific variation is high. We found that Caribbean reef sharks and nurse sharks occur in all waters surrounding the island, but Caribbean reef sharks were more common on the western side, whereas nurse sharks were more common on the northern side of the island. Furthermore, we found that the presence of Caribbean reef sharks in the shallow waters around Saba covered by the acoustic detection network differs throughout the day and across seasons, but also that intraspecific variation in both species may complicate generalizing monitoring results to population level for management purposes.

### 4.1. Ecology of Saba’s sharks

The Caribbean reef shark and nurse shark are the most common reef-associated shark species on many Caribbean reefs [16,17,37]. Our complementary approach shows that the abundance of the Caribbean reef sharks around Saba is 0.25 sharks per hour based on the stereo-BRUV approach and that divers have a mean probability of 56% of observing this species during a 1 h dive in the waters of Saba. For nurse sharks, this is 0.20 sharks per hour, with a probability of 25%. This abundance of Caribbean reef sharks was higher compared to Belize (0.04−0.13 sharks per hour [38]), and Sint Maarten (approx. 0.19 sharks per hour [39]), but similar compared to reefs of neighbouring Sint Eustatius and the Saba Bank [28,40].

The long-term observations through the citizen science program show that the probability of observing Caribbean reef sharks and nurse sharks significantly increased between 2012 and 2020. These results suggest that the abundance of both species around Saba may have increased during this period, which would be a reason for careful optimism about the management of these species around the island. Globally, reef-associated sharks continue to decline in abundance [16]. Currently, the Caribbean reef shark is listed as endangered, with populations throughout its range continuing to decline [1], whereas other populations in areas with more conservation measures (e.g. The Bahamas) are stable and show some annual increase [41]. The nurse shark is listed as vulnerable, with decreasing populations throughout its range, but the overall annual rate of change in the Northwest Atlantic shows a slight increase [1]. However, this significant trend in our diver-dependent data is likely influenced by other factors, such as the generally healthier reefs in the management zones where dives take place or increased culling of invasive lionfish by divers (i.e. consequential shark attraction/provisioning/habituation). We show that lionfish culling especially influences the number of Caribbean reef sharks observed during dives around Saba. This lionfish hunting may have caused sharks to be more attracted to divers as observed in other areas (e.g. [42]) thus potentially also contributing to an increase in diver-based observations as observed in this study. Although no long-term trends on lionfish culling exist for Saba, lionfish hunting has likely increased over the years as a response to increasing populations of invasive lionfish since 2009 [43]. However, these activities have not been logged consistently over the period of this study and lionfish culling by commercial dive schools on Saba only started after 2021. Prior to 2021, only marine park rangers were allowed to cull lionfish. Therefore, the increasing trends of shark observations presented in this study are likely impacted by other factors, may not represent population recovery and should be interpreted with caution.

Based on the length at maturity for both focal species [44], all except one Caribbean reef shark recorded by the BRUV systems were immature. This suggests that these insular habitats are important for the early life stages of this species, as has been suggested for other Caribbean reefs [28,45]. However, the BRUV survey did not include the pinnacles, whereas our telemetry results showed a high presence of Caribbean reef sharks at these pinnacles. This supports other studies that show that this (and other carcharhinid) species may experience ontogenetic shifts to deeper and more pelagic habitats [28,46,47].

The combined results of our telemetry and citizen science approaches show that both species showed year-round presence in Saba’s waters. On other Caribbean reefs, both Caribbean reef sharks and nurse sharks have been described to be present within the same area for similar or longer time periods [47–49]. Species closely related to the Caribbean reef shark are known to have high residency, with juvenile blacktip sharks (*Carcharhinus limbatus*) residing for up to 430 days in the same location and grey reef sharks (*Carcharhinus amblyrhynchos*) for up to 580 days [50,51]. Caribbean reef sharks showed high site fidelity to one location, although each shark, especially adults, visited multiple sites. Similar high site fidelity was measured for Caribbean reef sharks in the waters of Belize and Fernando de Noronha (Brazil) [38,45,52]. The site fidelity of nurse sharks in this study was relatively low compared to reefs in Belize [52], but the sample size for this species is small. Larger tracked distances (and lower site fidelity) of adult Caribbean reef sharks compared to juveniles might suggest an ontogenetic increase in home range as seen in other elasmobranch species [27,53,54]. For all nurse sharks, catch and release locations were not directly covered by detection stations. This could mean the sharks may have exerted high fidelity to sites outside the acoustic array or moved away from the area undetected. Nurse sharks in the waters of Belize were documented to have travelled an average of 7.7 km over reef habitats in 150 days [52]. However, other studies show that this species moves over longer (>900 km) distances [48].

Besides differences in movement ecology of life stages, it has been reported that adult male Caribbean reef sharks may stray more compared to female conspecifics [47]. The results of our acoustic telemetry study show that adult males were detected less, which could indicate a larger home range of these individuals and/or a higher rate of leaving Saba’s shallow waters, and may explain differences in detection probability across seasons [47]. Partial migration, in which a part of the population strays more compared to other conspecifics, has been recorded for other shark species and is suggested to contribute to gene flow and population connectivity [55,56]. Information on the dispersal range of a population can greatly improve the management of a species, as local MPAs covering a small area might be sufficient to conserve the resident part of a population [22,38,57]. However, for migrating or straying individuals, which likely warrant population connectivity between isolated or distant habitats, such local management efforts might not be as effective [22,57].

Our acoustic telemetry results show that the presence of Caribbean reef sharks is higher from December to May, which differs from the higher presence in The Bahamas during summer months [46], or from other studies that did not find evidence for the seasonality of this species [38,45,52]. This seasonal pattern coincides with the mating and parturition periods, which are considered to be between January and April for this species [58]. The presence of Caribbean reef sharks in the acoustic arrays was significantly higher at night compared to daylight hours, and based on the citizen science data, we show that nurse sharks were more frequently observed during night dives. These results agree with the findings of previous studies for both species that are probably linked to increased feeding activity [59–62]. Like Caribbean reef sharks, nurse sharks also move to shallower reefs and onto reef shelves during night hours to feed and to deeper reefs during daylight hours [62,63].

### 4.2. Complementary use of methods to inform shark management

We describe the complementary use of three conventional methods, stereo-BRUVs, citizen science observations and acoustic telemetry, to determine shark long-term spatiotemporal distribution and assess local species status. Each technique used answers different research questions or can be used to cross-validate outcomes from other methods and consolidate overall results (table 4).

**Table 4.** The different use of shark research methods related to different research topics for an effective complementary use (suitability of methods are indicated by: ‘--’ very poor, ‘-‘ poor, ‘+’ good, and ‘++’ very good).

ecological level	research topic	acoustic telemetry	citizen science	stereo-BRUVs
community	community composition (e.g. species diversity)	--	+	++
population/ species	population composition (e.g. life stages/sex ratio)	-	+	++
	long-term abundance trends	--	+	+
	residence and site fidelity	++	--	-
	long-term presence (e.g. seasonal/multi-annual)	++	++	+
	short-term presence (e.g. day/night)	++	+	+
	short-distance movement (e.g. habitat use)	++	+	++
	long-distance movement (e.g. (partial) migration)	+	--	-
individual	individual variation	++	--	-
stakeholder involvement	participation and contribution of stakeholders to research	+	++	+

Stereo-BRUV provides a short-term estimate of shark diversity, population composition (i.e. length frequency) and habitat use (table 4). This technique involves attracting sharks with bait, which could distort and bias the results of local fish abundance or short-distance movements [64,65]. In addition, around Saba, the stereo-BRUVs could not be deployed on the pinnacles—the location of the highest shark presence based on both acoustic telemetry and citizen science data—due to the potential damage to the fragile coral reefs covering the entire (small) surface of these pinnacles.

The citizen science data proved to be most valuable for determining long-term abundance trends of sharks within the study area [66,67], where a significant overall increase in the observation rate of both species on Saba’s reefs during 2012−2020 was found. Although citizen science data should be interpreted with caution due to misidentification of species, divers affecting shark behaviour, or overcounting of the same individuals [68], this dataset provided valuable means to validate results from the telemetry and stereo-BRUV approaches (e.g. [67]). In addition, the citizen science project allowed for an important stakeholder group (i.e. scuba dive companies) to be actively involved in research on sharks.

The shark telemetry component of this study provided valuable insights into the residence, fidelity and long-term movements of individual sharks in the waters of Saba. However, acoustic telemetry results can be confounded by disturbances in acoustic signals on complex habitat structures, wave action or turbidity and a large network of acoustic receivers is needed to cover long-distance movement patterns [69–71]. Furthermore, without the use of depth and/or activity sensors, discerning activity patterns and behaviour from acoustic telemetry data should be done with caution [47]. Therefore, combining this technique with video or diver surveys can improve the overall quality of the monitoring datasets.

High intraspecific variations in site fidelity, movement patterns and habitat use are common in shark species (e.g. [72]) and were also observed in this study. This variation, combined with limited sample sizes, complicates the generalization of results to population or species level and introduces uncertainty when designating temporal or area-based conservation measures. Acoustic tracking, as used in this study, can identify and account for this individual variation by following the same individuals for prolonged time periods. This highlights the importance of using animal tracking methods in combination with other monitoring techniques. However, the identification of individuals and accounting for individual variation in diver-operated and video surveys is possible with higher sampling effort and adaptation of these techniques [73,74]. Increasing the number of methods used to identify sources of this apparent variation can help discern if this is caused by specific groups within the population (e.g. adult males that stray more [47]) or if it is a true individual (and unpredictable) variation.

The complementary use of these three methods provided essential baseline data on the status of these reef-associated shark species, their high residence and fidelity to specific sites and habitats, their population structure and their seasonal and diurnal presence. We show that the regular and high observation rates of these species, their high residence and fidelity—especially of early life stages and females—highlight the importance of local area-based conservation measures. For these individuals, local area-based management efforts as part of the Yarari Marine Mammal and Shark Sanctuary may be sufficient. However, the seasonality in Caribbean reef shark presence and the low presence of adult male sharks also suggests that these species move over greater distances [47], probably outside protected waters to other reef systems in the region. It is these individuals that provide connectivity between distant shark populations and ecosystems [22,56], and their movements potentially require a larger network of MPAs [26,57]. Overall, the complementary use of acoustic telemetry, stereo-BRUVs and a citizen science (scuba) program provides a valuable baseline assessment of shark diversity, presence and distribution for the delineation of ecologically important sites (e.g. Important Shark and Ray Areas [75]). Therefore, these methods can be used to collect baseline information for effective area-based conservation of reef-associated shark species, especially in the relatively understudied Dutch Caribbean waters.

## Data Availability

The data used in this study can be accessed through the repository [76].
